# From contamination to infective endocarditis—a population-based retrospective study of *Corynebacterium* isolated from blood cultures

**DOI:** 10.1007/s10096-019-03698-6

**Published:** 2019-09-04

**Authors:** Magnus Rasmussen, Anna Wramneby Mohlin, Bo Nilson

**Affiliations:** 1 Section for Infection Medicine, Department of Sciences Lund, Lund University, Lund, Sweden; 2 Division for Infectious Diseases, Skåne University Hospital, Lund, Sweden; 3 Division of Infection Medicine Diseases, Department of Clinical Sciences Lund, Lund University, BMC B14, SE-223 63 Lund, Sweden; 4 Department of Clinical Microbiology, Labmedicin, Region Skåne, Lund, Sweden; 5 Division of Medical Microbiology, Department of Laboratory Medicine Lund, Lund University, Lund, Sweden

**Keywords:** *Corynebacterium*, Bacteremia, Infective endocarditis, Contamination, Blood culture

## Abstract

*Corynebacterium* is a genus that can contaminate blood cultures and also cause severe infections like infective endocarditis (IE). Our purpose was to investigate microbiological and clinical features associated with contamination and true infection. A retrospective population-based study of *Corynebacterium* bacteremia 2012–2017 in southern Sweden was performed. *Corynebacterium* isolates were species determined using a matrix-assisted laser desorption/ionization-time-of-flight mass spectrometry (MALDI-TOF MS). Patient were, from the medical records, classified as having true infection or contamination caused by *Corynebacterium* through a scheme considering both bacteriological and clinical features and the groups were compared. Three hundred thirty-nine episodes of bacteremia with *Corynebacterium* were identified in 335 patients of which 30 (8.8%) episodes were classified as true infection. Thirteen patients with true bacteremia had only one positive blood culture. Infections were typically community acquired and affected mostly older males with comorbidities. The focus of infection was most often unknown, and in-hospital mortality was around 10% in both the groups with true infection and contamination. *Corynebacterium jeikeium* and *Corynebacterium striatum* were significantly overrepresented in the group with true infection, whereas *Corynebacterium afermentans* was significantly more common in the contamination group. Eight episodes of IE were identified, all of which in patients with heart valve prosthesis. Six of the IE cases affected the aortic valve and six of seven patients were male. The species of *Corynebacterium* in blood cultures can help to determine if a finding represent true infection or contamination. The finding of a single blood culture with *Corynebacterium* does not exclude true infection such as IE.

## Introduction

*Corynebacterium* is a genus of Gram-positive rods comprising more than 100 species of which a large proportion has been associated to human infections [1]. *Corynebacterium diphtheriae* is the most well-known of these species, but over the last decades, more attention has been drawn to non-diphtheriae *Corynebacterium* species as human opportunistic pathogens [1].

When *Corynebacterium* is isolated from blood cultures, the finding is often dismissed as a contamination from normal skin flora rather than recognized as a pathogen causing true infection. Studies investigating different cohorts have indicated that between 44 and 71% of patients with *Corynebacterium* bacteremia have true infection [2–4]. Some studies have only used a bacteriological criterion (≥ 2 positive blood cultures) [2, 3] whereas other studies also considered clinical features and acknowledge that intravascular devices are risk factors for true *Corynebacterium* infection [4–6]. The lack of a uniform definition of contamination and infection hampers the possibility to draw conclusions about the incidence of true *Corynebacterium* infections. Moreover, available studies describe limited number of cases and have a retrospective design [2–5, 7].

*Corynebacterium* can cause infective endocarditis (IE) and is a rare etiology of such infections [8]. Knowledge on *Corynebacterium* IE comes mainly from case reports or systematic reviews of the literature. From a large systematic review it was concluded that *Corynebacterium* IE mainly affects the left side of the heart (95% of cases) and that men constituted 72% of described cases [9]. In this case review, 19% of cases represented valve prosthesis IE and the mortality rate was high (40%) [9]. Interestingly, a difference in clinical presentation between different species of corynebacterial was noted [9].

Species determination of corynebacteria has been challenging [1] but the introduction of matrix-assisted laser desorption/ionization-time-of-flight mass spectrometry (MALDI-TOF MS) has provided a practical tool for this purpose [10, 11]. It has been shown that species determination of *Corynebacterium* can be performed with sufficient precision using improved databases and lower cutoff scores in the analysis of spectra [11]. Several studies have investigated the features of infections with specific *Corynebacterium* species. Of non-diphtheriae corynebacteria, *Corynebacterium striatum* and *Corynebacterium jeikeium* have often been reported as pathogens [3, 5, 7, 9, 12]. However, at present, there is insufficient data on how species determination of corynebacteria can help to determine the risk for true infection or even IE in patients with *Corynebacterium* bacteremia.

The present study aims to determine risk factors for true *Corynebacterium* infection, including IE, in a large retrospective population-based cohort of patients with positive blood cultures.

## Methods

### Microbiology and species determination

Data on patients with blood cultures positive for *Corynebacterium* were collected from the registry of the Laboratory for Clinical Microbiology in the county of Skåne, Sweden, for the years 2012 to 2017. A blood culture was regarded to be positive irrespective of if one or two bottles yielded growth. The laboratory is the only one in Skåne, a province with a population of 1,329,000 inhabitants (January 1, 2017, data from Statistics Sweden (available at https://www.scb.se), and all cultures from this province (including nine hospitals and all primary care facilities) are handled by our laboratory. From 2012 through late 2014, the BacT/Alert blood culture system (bioMérieux, Marcy l’Etoile, France) was used and was replaced by the BACTEC FX blood culture system (Becton Dickinson, Franklin Lakes, USA) in December 2014. The laboratory receives approximately 75,000 blood culture bottles/year of which roughly 12% grow bacteria. The isolates had been identified by MALDI-TOF MS and the majority of them were identified at genus level only. For the study, stored isolates were re-cultured on blood agar plates under aerobic conditions, and when sufficient growth was observed (at between 24 and 120 h), samples were analyzed by the direct colony method using MALDI-TOF MS (version Microflex MALDI-TOF MS, software FlexControl 3.4 and MALDI Biotyper (MBT) Compass 4.1, with MBT Compass Library, DB-7854 MSP (Bruker, Bremen, Germany). In cases where a low score was achieved, we also used a standard ethanol-formic acid extraction method described by the instrument manufacturer. The cutoff of 1.7 for species determination as suggested by Alatoom et al. [11] and also others [13] was employed. Resistance was determined according to EUCAST protocols, and breakpoints were defined according to EUCAST guidelines (available at EUCAST.org).

### Patients and definitions

Medical records were studied retrospectively. Records inaccessible and patients under the age of 18 were excluded from further analysis. The regional ethics committee of Lund University approved of the study (2017/1002). An episode was defined as a clinical situation where *Corynebacterium* was isolated from blood and the episode was concluded after 2 weeks of effective antibiotic treatment. Thus, if *Corynebacterium* was isolated from the same patient within 14 days, this was regarded as persistent bacteremia whereas a positive culture after 14 days was regarded as a new episode. Data collected included age, gender, the use of immunosuppressive treatment, underlying medical conditions according to Charlson [14], and criteria for IE according to Duke [15]. Nosocomial infections were defined as an infection evident at 48 h or more past hospital admission. Focus of infection was defined by fulfillment of the following three criteria (i) the isolation of the relevant bacterium from the site of infection, (ii) signs and symptoms of focal infection, and iii) radiological signs of focal infection [16]. In-hospital mortality was recorded.

We modified the definitions for discrimination of true infection from contamination described previously by Finkelstein and co-workers [6] and presented our definition in Table 1. For episodes with two or more blood cultures with growth of *Corynebacterium*, signs of infection (one of the following: temperature ≥ 38 °C, systolic blood pressure < 100 mmHg, presence of chills or leukocytosis (> 12 × 10^9^/L)) were needed for the episode to be classified as true infection (criterion 1). True infection was rejected if another more likely cause of the infection, such as a positive blood culture with more pathogenic bacteria (criterion 2) or a focal infection where another bacterial species was deemed to be a more likely cause (criterion 3). If another bacterium was isolated from the site of infection or if the focal infection was pneumonia, soft tissue infection, or urinary tract infection, this excluded true *Corynebacterium* infection*.* If the *Corynebacterium* was isolated from the site of infection or if the focal infection was IE, spondylitis, and arthritis with no findings of bacteria from the focal infection, *Corynebacterium* was regarded as the most likely etiology. To classify episodes with only one blood culture with *Corynebacterium* as true infection, the episode had to fulfill criteria 1–3, and in addition, an intravascular device had to be at place for more than 48 h or *Corynebacterium* had to be isolated from the site of infection. Episodes of *Corynebacterium* bacteremia not fulfilling above criteria were regarded as contamination.

**Table 1:** Definitions of true infection depending on the number of positive cultures

Two or more positive blood cultures	
Criterion 1	Infection confirmed by temperature > = 38 OR hypotension (systolic blood pressure < 100 mmHg) OR chills OR leukocytosis (> 12 × 10^9^/L)
Criterion 2	No other more likely pathogen in blood culture explains confirmed infection
Criteria 3	No other focal infection where other bacteria are more likely explains symptoms of patient, where focal infection is defined by two of the following (a–c): a. Isolation of pathogens other than *Corynebacterium* at site of infection b. Typical signs or symptoms of focal infection c. Imaging results compatible with focal infection
Additional criteria in cases with one positive blood culture	
Criteria 4	Foreign intravascular device^a^ present > 48 h prior to blood sample OR *Corynebacterium* isolated at site of infection

^a^Central venous catheters, port-á-cath, implantable cardioverters, pacemakers, prosthetic heart valves, intravascular grafts, and picc-lines

### Statistical analysis

For categorial data, statistical analysis was performed using two-sided *χ*^2^ test (Pearson) for multiple comparisons or Fischer’s exact test for pairwise comparisons. Continuous variables were investigated for difference using Mann-Whitney *U* test. A *p* value of < 0.05 was regarded as statistically significant.

## Results

### Description of the cohort

Four hundred twelve blood cultures with growth of *Corynebacterium* were identified in 369 patients with 373 episodes of bacteremia. Ten episodes were excluded because of inaccessible medical records and 24 episodes because of patient age under 18 years. This resulted in a total of 335 patients with 339 episodes in the final analysis. Thirty (8.8%) episodes were classified as true infection, whereas 309 (91%) episodes were classified as contamination. Characteristics of the clinical features of episodes are listed in Table 2. Both groups were predominantly elderly males with comorbidities. *Corynebacterium* was cultured (*n* = 6) or detected by 16S rRNA gene PCR and DNA sequencing (*n* = 3) from the site of focal infection in nine episodes in which four patients had IE, and one each had spondylodiscitis, joint prosthesis infection, septic arthritis, pneumonia, central-line infection, and urinary tract infection.

**Table 2:** Characteristics of patients with true infection and contamination

	True infection (*n* = 30)	Contamination (*n* = 309)	*p* value for difference
Age years (IQR^1^)	74 (69–80)	75 (63–83)	1
Female gender, *n* (%)	9 (30)	129 (38)	0.2
Charlson score			
0–1	7 (23)	129 (42)	0.1
2–3	12 (40)	105 (34)	
≥ 4	11 (37)	75 (24)	
Immunosuppressive treatment	5 (17)	31 (10)	0.3
Intravascular device	20 (67)	37 (12)	< 0.0001
Community site of acquisition	25 (83)	278 (90)	0.3
Focus of infection			
Unknown	19 (63)^2^	165 (53)^3^	ND^4^
IE	5 (17)	0	
Bone and joints	3 (10)	3 (1.0)	
Lungs	1 (3.3)^5^	63 (20)	
Urinary tract	1 (3.3)^5^	24 (7.8)	
Central-line	1 (3.3)^5^	0	
Skin/soft tissue	0	28 (9.1)	
Abdominal	0	20 (6.5)	
Other	0	6 (1.9)	
In-hospital mortality	3 (30)	33 (11)	1

^1^Inter-quartile range. ^2^Includes three episodes of possible IE. ^3^Unknown focus or no infection. ^4^Not determined due to too few observations in some cells. ^5^*Corynebacterium* isolated at the site of infection

### Identification and characterization of isolates

Two hundred fifty-eight of 339 (76%) isolates could be classified to the species level; remaining isolates had either not been saved or could not be recultured from stocks. The most abundant species identified in the episodes representing true infections and contaminations are given in Table 3. *Corynebacterium afermentans* was the most commonly isolated species in episodes representing contamination, whereas *C. striatum* was the most common species in cases of true infections. Despite its low frequency in the microbiological data set, four of seven episodes with *C. jeikeium* were categorized as true infection. The difference in species distribution between the groups was statistically significant (*p* < 0.0001 in chi-square test for difference).

**Table 3:** Microbiology of true infections and contamination

	True infection (*n* = 30)	Contamination (*n* = 309)	*p* for difference
Two or more positive blood cultures	17 (57)	25 (8.1)	< 0.0001
Polymicrobial	4 (13)^1^	97 (31)	0.04
Species of *Corynebacterium*, *n* (%)			
Not determined to species	4 (13)	81 (25)	< 0.0001
*C. striatum*	8 (27)	34 (11)	
*C. jeikeium*	4 (10)	3 (1)	
*C. aurimucosum*	3 (10)	20 (6.5)	
*C. amycolatum*	3 (10)	16 (5.2)	
*C. afermentans*	0	46 (15)	
Other species	8 (27)^2^	109 (37)^3^	

^1^The other bacterium was a coagulase-negative *Staphylococcus* (*n* = 2), a *Micrococcus* (*n* = 1), and *Cutibacterium acnes* (*n* = 1). ^2^Other species were *C. propinquum* (*n* = 2), *C. pseudodiphtericum* (*n* = 1), *C. glucoronolyticum* (*n* = 1), *C. mucifaciens* (*n* = 1), *C. riegelii* (*n* = 1), *C. stationis* (*n* = 1), and *C. ulcerans* (*n* = 1). ^3^*C. minutissimum* (*n* = 15), *C. imitans* (*n* = 11), *C. lipophiloflavum* (*n* = 9), *C. tuberculostearicum* (*n* = 9), *C. coyleae* (*n* = 8), *C. propinquum* (*n* = 7), *C. mucifaciens* (*n* = 7), *C. singulare* (*n* = 7), *C. pseudodiphthericum* (*n* = 6), *C. glucoronolyticum* (*n* = 6), *C. simulans* (*n* = 4), *C. riegelii* (*n* = 4), *C. stationis* (*n* = 3), *C. macginleyi* (*n* = 2), *C. xerosis* (*n* = 1), *C. falsenii* (*n* = 1), *C. resistens* (*n* = 1), *C. glaucum* (*n* = 1), *C. epidermidicanis* (*n* = 1), *C. kroppenstedtii* (*n* = 1), *C. durum* (*n* = 1), *C. ureicelerivorans* (*n* = 1), *C. urealyticum* (*n* = 1), *C. ammoniagenes* (*n* = 1), and *C. pyruviciproducens* (*n* = 1)

Of isolates from true infections, the majority were resistant to penicillin and clindamycin (25 and 24 of 30 tested isolates, respectively), whereas a majority of isolates were sensitive to fluoroquinolones and rifampicin (13 of 24 and 18 of 21 isolates, respectively). All isolates tested were sensitive to vancomycin (*n* = 30, median MIC 0.5) and linezolid (*n* = 22).

### IE caused by *Corynebacterium*

Eight episodes of bacteremia were regarded and treated as IE (see Table 4 for clinical descriptions). *C. jeikeium* and *C. striatum* were responsible for two cases each whereas *C. propinquum*, *C. amycolatum*, and *C. pseudodiphtericum* were responsible in one patient (of whom one had two episodes) each. In five cases, Duke’s criteria for definite IE were fulfilled, and in one patient with possible IE, ^FDG^PET-CT showed strong uptake in relation to the valve prosthesis. Interestingly, all cases were valve prosthesis IE, of which six affected the aortic valve and six of seven patients were male. The time from valve insertion to the episode of IE varied from 4 weeks to 3 years. One patient died from the infection; one patient had a recurrent IE; and, in five cases, valve surgery was performed.

**Table 4:** Characteristics of IE caused by *Corynebacterium*

Gender	Age	Species	No. of blood cultures	Valve	TEE finding	Diagnosis	Treatment	Outcome	Comment
Male	66	C. *striatum*	3/3	BAP^1^	Veg, abscess	Def	New BAP	Death	PCR on valve pos
Female	60	C. *jeikeium*	2/2	BAP	Abscess	Def	Homograft	Cured	PCR on valve pos
Male	56	C. *striatum*	2/2	BAP	Suspect	Poss	Conservative	Cured	
Male	75	C. *jeikeium*	1/3	BAP	Veg	Def	Homograft	Cured	PCR on valve pos CNS embolism
Male	70	*C. propinquum*	8/8	BAP	Normal	Poss	Conservative	Cured	
Male	69	C. *amycolatum*	2/4	BAP, BMP	MI	Def	Conservative	Relapsed	
Male	69	C. *amycolatum*	4/4	BAP, BMP	Veg	Def	New BMP	Cured	PCR on valve pos CNS embolism
Male	79	C. *pseudo-diphtheriticum*	2/3	BAP	Normal	Poss	Homograft	Cured	PET-CT pos

^1^*BAP*, biological aorthic prosthesis; *veg*, vegetation; *def*, definite IE according to Duke; *poss*, possible according to Duke; *BMP*, biological mitral prosthesis

## Discussion

This work demonstrates that *Corynebacterium* is a rare cause of severe infections and that bacteremia often represent contamination. Since our cohort is population-based, we conclude that the incidence of *Corynebacterium* IE is around one in a million per year, whereas true bacteremia occurs in around four cases per million inhabitants per year. On several occasions, very significant infections such as culture-proven *Corynebacterium*-caused IE or spondylodiscitis were diagnosed in patients with only one positive blood culture. This indicates that the definitions used for differentiating between true infection and contamination should take into account both bacteriological and clinical factors. Simple definitions such as one blood culture equals contamination will miss true infections. Our definition included a demand on clinical symptoms of infection, lack of other more likely causes of infection, and either two positive blood cultures or one positive blood culture in conjunction with an intravascular device or isolation of *Corynebacterium* at the site of a focal infection. This was adapted from previous works on coagulase-negative staphylococci [6] which, similar to *Corynebacterium*, often contaminate blood cultures and on rare occasions cause severe infections. Our definition is more sensitive than simple microbiological definitions based only on the number of positive cultures but there is also a risk for introducing false positives. Some of the episodes where *Corynebacterium* was isolated from two blood cultures and no focus was identified could certainly also represent contamination, whereas some likely represent missed cases of IE. We believe, however, that a more inclusive definition of true infection is important in order not to miss severe infections caused by *Corynebacterium*.

From our results, we conclude that the species of *Corynebacterium* is an important factor in determining if a finding is likely to represent true infection or contamination. Thus, the finding of *C. jeikeium* in blood is a strong suggestion of an underlying true infection whereas the isolation of *C. afermentans* strongly indicates contamination. We therefore suggest that clinical laboratories should determine the species of all corynebacterial isolated from blood so that clinicians can use this information to determine if a given isolate is a contaminant or relevant pathogen.

*Corynebacterium* IE has been described in numerous case reports but large case series or cohort-based studies are lacking. Our description of eight episodes of *Corynebacterium*-caused IE is relevant as it represents a population-based account of this type of infection. Interestingly, all cases were related to valve prosthesis and this is clearly more than reported in a large review of published cases (19%) [9]. The cases occurred both early after valve insertion and as late as 3 years after surgery suggesting that both intraoperative and hematogenous spread of bacteria occur. Our findings also demonstrate that the isolation of a *Corynebacterium* from blood in a patient with a heart valve prosthesis should evoke a strong suspicion of IE. Despite the potential severity of prosthesis-IE, the mortality was lower in our cohort (one of seven patients) than that reported from systematic reviews of case reports (40%) [9]. This might be explained by a tendency of clinicians to mainly report dramatic presentations of IE. As in previous reviews on the topic, we observed a male dominance in *Corynebacterium* IE. Despite that our cohort is comparatively large, it is still too small to draw definite conclusions about the role of different *Corynebacterium* species in IE. It can be noted, however, that *C. amycolatum*, *C. striatum*, and *C. jeikeium* constitute a majority of cases and these species have been described previously to be common in *Corynebacterium* IE.

*C. striatum* has recently been shown to possess virulence factors, especially in terms of biofilm production, which implies that favorable conditions for the bacteria such as venous catheters constitute a risk environment for the growth of this particular bacterial species [17]. Indeed, spread and adaption of certain *C. striatum* clones in hospital settings has been demonstrated recently [18]. *C. striatum* is also known as a causative agent of a wide range of infections and for displaying or easily develop resistance to many antimicrobials [19–22]. Apart from IE and sepsis, *C. striatum*–related infections can arise from the respiratory tract, urinary tract, or wounds [23]. This is consistent with the findings in this study, where *C. striatum* was related to different types of infections.

The main strength of this study is that it is population-based and large. This allows for conclusions about incidences. Another strength is the combination of microbiological analyses and a careful analysis of clinical data which allow us to draw conclusions about the likelihood of different species to cause infection and contamination. The main limitation is that not all isolates were available for species determination and that there was a bias in which isolates had been saved. Isolates that the laboratory staff believed represented contaminations were less likely to be preserved, and this might have skewed the results from the species determination. The study was of course also limited by the retrospective design where only information recorded in the medical records were available for analysis. Despite that the study is population-based, of course, caution must be taken with extrapolation of the results to other geographical locations.

In conclusion, we demonstrate that *Corynebacterium* can cause severe infections and that species determination is an important tool to help clinicians in determining if a given patient has true infection caused by *Corynebacterium*. Therefore, we propose that microbiology laboratories should report the species of corynebacteria isolated from blood cultures.
